# Selection for early shoot vigour in wheat increases root hair length but reduces epidermal cell size of roots and leaves

**DOI:** 10.1093/jxb/erac048

**Published:** 2022-02-23

**Authors:** Pieter-Willem Hendriks, Peter R Ryan, Philip Hands, Vivien Rolland, Saliya Gurusinghe, Leslie A Weston, Greg J Rebetzke, Emmanuel Delhaize

**Affiliations:** 1 CSIRO, Agriculture and Food, Canberra, ACT, 2601, Australia; 2 Charles Sturt University, School of Agriculture, Environment and Veterinary Sciences, Wagga-Wagga, 14 NSW, 2650, Australia; 3 Graham Centre for Agricultural Innovation, Wagga Wagga, NSW, 2678, Australia; 4 Australian Plant Phenomics Facility, Australian National University Node, 134 Linnaeus Way, Acton ACT 2601, Australia; 5 University of Antwerp, Belgium

**Keywords:** Cell body, cell size, competitiveness, early shoot vigour, rhizosheaths, root hairs, selection, trichoblast, wheat

## Abstract

Six cycles of recurrent selection for early shoot vigour in wheat resulted in significant increases in leaf width and shoot biomass. Here, in replicated controlled-environment studies, the effect of early shoot vigour on root biomass, rhizosheath size, root hair length, and cell size in the roots and leaves was examined across different cycles of selection. Increased shoot vigour was associated with greater root biomass, larger rhizosheath size, and longer root hairs. Our findings demonstrate that rhizosheath size was a reliable surrogate for root hair length in this germplasm. Examination of the root epidermis revealed that the ‘cell body’ of the trichoblasts (hair-forming cells) and the atrichoblasts (non-hair-forming cells) decreased in size as shoot vigour increased. Therefore, in higher vigour germplasm, longer root hairs emerged from smaller trichoblasts so that total trichoblast volume (root hair plus cell body) was generally similar regardless of shoot vigour. Similarly, the sizes of the four main cell types on the leaf epidermis became progressively smaller as shoot vigour increased, which also increased stomatal density. The relationship between shoot vigour and root traits is considered, and the potential contribution of below-ground root traits to performance and competitiveness of high vigour germplasm is discussed.

## Teaser

The selection for early shoot vigour in wheat is associated with increased root hair length and changes to cell size in roots and shoots.

## Introduction

Early vigour in wheat (*Triticum aestivum* L.) is exemplified by rapid canopy growth, resulting in increased soil coverage (Richards, 1989), and is positively correlated with the length, width, and area of the first leaves (Rebetzke and Richards, 1999; Maydup *et al.*, 2012). Leaf area and shoot biomass are factors that positively influence the ability of wheat to compete with weeds because they prevent competing species from accessing sunlight (Gaudet and Keddy, 1988). Plant height is also considered a beneficial trait for improving competitiveness as it is a major driver of above-ground biomass (Lemerle *et al.*, 1996). However, during the Green Revolution of the 1960s, the average height of wheat crops was reduced by the introduction of dwarfing genes, *Rht-B1b* and *Rht-D1b*, to improve the harvest index and reduce lodging (Murphy *et al.*, 2008). The resulting semi-dwarf cultivars exhibited reduced early vigour (Rebetzke *et al.*, 2004) and lower shoot biomass, and were less competitive than their taller progenitors (Vandeleur and Gill, 2004). Considerable variation in early shoot vigour was identified among the semi-dwarf varieties, and additional selection for vigour generated modest increases in competitiveness without compromising the harvest index (Zerner *et al.*, 2016). A focused breeding programme for early shoot vigour is thus expected to further enhance weed competitiveness (Rebetzke *et al.*, 2018).

An intrapopulation breeding programme in wheat based on recurrent selection for early shoot vigour using a diverse set of globally sourced vigorous germplasm was described by Zhang *et al.* (2015). Progeny showing enhanced leaf width were selected and randomly intercrossed. This procedure was repeated over six cycles spanning 15 years, resulting in a near-linear increase in leaf width, leaf area index, and shoot biomass with each cycle of selection. While the positive impact of early shoot vigour on weed competitiveness is well established (Bertholdsson, 2005), the relationship between shoot vigour and other traits, including below-ground architecture and root development, is unclear. Further insight into whether the drivers of shoot vigour are restricted to the shoots or reflect a widespread change to plant development is required. It is unknown whether shoot vigour is linked with specific root traits that might also contribute to the enhanced competitiveness of that germplasm. After all, prior to canopy closure, the competition for below-ground resources is likely to be more intense than competition above-ground (Wilson, 1988; Kiær *et al.*, 2013). Casper and Jackson (1997) also pointed out that ‘where’ and ‘when’ roots are deployed may be as important as average root density in explaining below-ground competitiveness.

Wheat cultivars with more vigorous shoots tend to possess larger root systems (Palta *et al.*, 2011; Palta and Yang, 2014). One study demonstrated that the mature root systems of ‘vigorous’ wheats accumulated up to 66% more carbon than less vigorous commercial cultivars (Palta and Watt, 2009) and are more efficient at accessing nitrogen and phosphorus from the soil (Liao *et al.*, 2004; Ryan *et al.*, 2015). The effect of shoot vigour on other root traits, including root hair length and cell size, have not been investigated in detail, even though they could explain plant performance and reveal clues to the developmental mechanisms driving increased plant vigour.

Root hairs are likely to be associated with increased crop competitiveness as well. These protrusions from the trichoblasts on the root epidermis (Libault *et al.*, 2010; Dolan, 2017) increase the volume of soil explored by the roots and enhance surface area required for absorption of water and nutrients (Passioura, 1988). Along with fine roots, root hairs enhance a plant’s ability to access critical soil resources for a relatively low carbon cost (Hutchings and de Kroon, 1994; Datta *et al.*, 2011; Brown *et al.*, 2012; Comas *et al.*, 2013; Rongsawat *et al.*, 2021). Root hair length, density, and distribution impact the plant’s ability to access resources (Gilroy and Jones, 2000; Ruiz *et al.*, 2019), particularly in challenging environments (Carminati *et al.*, 2017; Vincent *et al.*, 2017). For example, a study with root hairless barley mutants demonstrated that under drought conditions the absence of root hairs reduced water and nutrient uptake by 3-fold (Marin *et al.*, 2021). Indeed, some consider the volume of soil occupied by roots to be more important for competitiveness than the total size of the root system (Craine and Dybzinski, 2013). Interestingly, wheat exhibits variation in root hair length and density, and cultivars with long root hairs may also exhibit greater hair density (Gahoonia *et al.*, 1997). Apart from increased soil exploration and nutrient absorption, root hairs may contribute to below-ground competitiveness through other rhizospheric interactions including the exudation of secondary metabolites that influence the growth of competing plants, or allelopathy (Holz *et al.*, 2018). It has been suggested that plants with long, dense root hairs exhibit enhanced allelopathic potential (Bertin *et al.*, 2003) by increased production and release of bioherbicides that suppress the growth of competing species (Weston, 2005; Weston *et al.*, 2012).

Given the lack of information on the relationship between shoot vigour and below-ground development in wheat, we examined specific root traits in germplasm generated from the recurrent selection for increased shoot vigour. Specifically, we assessed root epidermal and leaf epidermal cell sizes to test the hypothesis that enhanced shoot vigour alters the number and size of cells in these organs. We also measured rhizosheath size as a proxy for root hair length (Delhaize et al., 2012, 2015) given that root hair assessments are particularly challenging (Watt *et al.*, 1994; Pang *et al.*, 2017). The hypothesis that trichoblast cell body size is associated with root hair length was also tested. We discuss how root traits could be contributing to the superior competitiveness of vigorous germplasm.

## Materials and methods

### Germplasm

Experiments were performed with wheat genotypes developed by recurrent selection for high shoot vigour in an intrapopulation wheat breeding programme (Zhang *et al.*, 2015). Briefly, globally sourced wheat genotypes exhibiting high shoot vigour were randomly crossed to generate the first cycle. Progeny of these crosses showing the greatest vigour, as measured by leaf width, were selected and progeny-tested. Lines demonstrating greatest vigour were then intercrossed to generate the germplasm for the next cycle. Six cycles of selection were completed from Cycle 0 (C0, least vigorous) to Cycle 6 (C6, most vigorous), each of which included ~30 lines (Zhang *et al.*, 2015). The lines examined in the present study were randomly sampled from Cycle 0 (C0), Cycle 1 (C1), Cycle 3 (C3), Cycle 4 (C4), Cycle 5 (C5), and Cycle 6 (C6). The numbering of these lines followed the nomenclature of Zhang *et al.* (2015), and the individual lines tested in this study from each cycle were as follows: C0 (lines 1, 6, 8, 10, and 17), C1 (lines 1, 2, 3, 11, 14, and 15), C3 (lines 1, 5, 11, 28, and 36), C4 (lines 17, 19, 21, 32, and 37), C5 (lines 4, 28, 31, 36, and 39), and C6 (lines 2, 26, 38, 39, and 54). Seed was obtained from plants that were grown together under the same optimal growing conditions in a glasshouse and stored at –18 °C until the start of the experiments.

### Soils

As soil is a complex growth medium and its physical structure and composition interact with root growth (Bengough, 2003), two contrasting agricultural field soils were used in all rhizosheath growth experiments. Soil 1 was a Podzol obtained from the ‘Wallaroo’ site at the Ginninderra Experimental Station (35°17ʹS, 149°05ʹE; ACT Australia). Here, the topsoil was collected to a depth of 10 cm and was acidic (pH 4). The soil was limed with CaCO_3_ (2 g kg^–1^ of soil) to achieve a pH of 7. Soil 2 was a Red Kandosol collected from the Graham Centre Field in Wagga Wagga (35°03ʹS, 147°36ʹE; NSW Australia). The topsoil (0–10 cm) had a pH of 4.9 that had been previously limed with CaCO_3_ (1.5 g kg^–1^ of soil) to pH 7. Both soils were sieved through a 4 mm mesh and three random subsamples were oven-dried to determine soil moisture content. Deionized water was added to each soil to reach 50% field capacity at ~11% moisture content. For rhizosheath assays, the soils were placed in pots of volume 192 cm^3^ (40 × 40 × 120 mm) at a bulk density of 1.1 g cm^–3^. The rhizosheath experiment was replicated twice in the Podzol and once in the Red Kandosol.

### Seedling growth

To minimize maternally induced variation among the genotypes examined (Evans and Bhatt, 1977; Peterson *et al.*, 1989), all experimental comparisons were made on seedlings and plants grown from seeds of similar weight (47 ± 1 mg). Seed was treated with fungicide by soaking for 3 min in a Thiram® (1.4 g l^–1^) solution. Eight to 10 seeds of each genotype and replication were placed on wet filter paper in Petri dishes incubated at 4 °C for 3 d for germination at ambient temperature (~25 °C) for 24 h. Seedlings with roots between 3 mm and 7 mm long were selected to be sown. Transplanted seedlings were then placed in a growth cabinet with a 12/12 h day/night regime at 20/15 °C day/night and a daytime light intensity of 600 μmol m^−2^ s^−1^.

### Shoot vigour assessment

For assessment of shoot vigour, six seedlings for each genotype from the cycles of recurrent selection were sown in opaque black pots (4 litres, 200 mm diameter, 190 mm height, ANOVApot®). Seedlings were sown vertically and spaced evenly in the pot filled with a soil mix (50% river sand/50% potting compost v/v). Pot position was randomized in growth cabinets (12/12 h day/night regime with temperatures set at 20/15 °C day/night, and daytime light intensity of 600 μmol m^−2^ s^−1^). The plants were harvested at leaf stage 4 (Z14) (Zadoks *et al.*, 1974), and the widths of the first, second, and third leaves were assessed using a digital calliper. Roots and shoots were separated and dried for 72 h at 70 °C before weighing, and the root to shoot ratios of the various lines were calculated. The experiment, designed as a complete randomized block, was replicated four times.

### Rhizosheath and root hair imaging and measurements

**Fig. 1.**
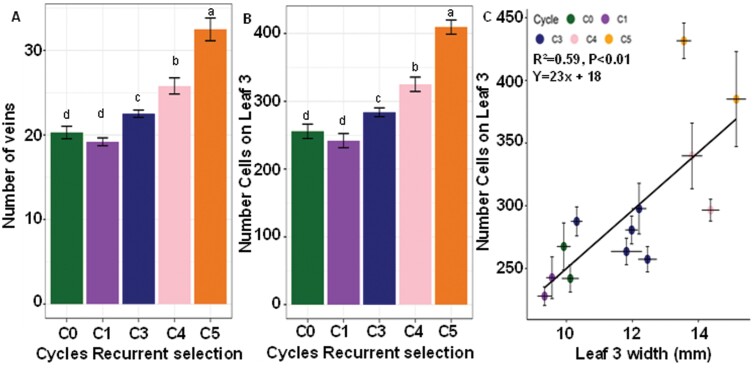
Number of veins and cell files across the leaves of C0 to C5 lines. Data show (A) the number of veins across the width of the third leaf, (B) the number of cell files over the width of the third leaf of the cycles for recurrent selection, and (C) the relationship between the width of leaf 3 and number of cell files. The error bars represent SEs (*n*=15 for vein and cell file numbers and *n*=24 for leaf width). Letters identify significant differences between means (*P*≤0.05).

In each rhizosheath experiment, six seedlings per genotype were grown as described above under the heading ‘Seedling growth’. After 8 d, plants were gently removed from the pots so that the residual soil remained attached to the roots. The first three seminal roots were excised at the seed, and root length and fresh weight were measured (root plus soil) (Delhaize *et al.*, 2012). Rhizosheath size is expressed as the ratio of the fresh weight to length of the three seminal roots. The roots were then washed to remove soil and stored in a 50% (v/v) ethanol solution. A 1 cm segment was excised from the middle of the first seminal root that had been removed from each plant, placed on a glass slide with 50% ethanol, and gently agitated to ensure root hairs were evenly spread from the epidermis. After covering with a glass coverslip, samples were observed using a Leica M205c® dissecting microscope equipped with a ×1.0 objective and light-emitting diode ring illumination, and imaged using a Leica IC90e® camera and Leica Application Suite V4.12 (LAS-X, Leica Microsystems; Fig. 1). Root hair length was estimated using the FIJI image processing package (version 1.53c) (Schindelin *et al.*, 2012). The longest root hair was measured on both sides of the roots such that a total of 10 assessments were taken for each root sample as described previously (Delhaize *et al.*, 2012).

### Root cell measurements

Root epidermal cells were measured using tissues stored in 50% (v/v) ethanol that had been previously used to measure rhizosheath size and root hair length. Five 1 cm length root samples were excised from seedlings generated for all genotypes of the six recurrent selection cycles. Tissues were rehydrated by treatment with 25% ethanol (v/v) for 5 min followed by two deionized water rinses of 5 min using gentle agitation followed by staining in 0.1% (w/v) acridine orange (Sigma-Aldrich) in 5% (w/v) acetic acid for 2 min under a vacuum of ~0.07 MPa. Samples were rinsed twice in fresh distilled water for 2 min with gentle agitation, and mounted in water using glass slides and 0.17 mm thick coverslips.

Confocal images were obtained using a Leica SP8 confocal laser‐scanning microscope (Leica Microsystems, Australia) equipped with a ×20 (numeric aperture=0.5) water immersion objective and the Leica Application Suite V3.5 (LAS-X, Leica Microsystems). Images were collected with 488 nm excitation and 520–580 nm emission. Image *z*-stacks sufficient to capture the entire depth of the epidermal cells for each sample were collected using a 2.05 µm step size, this being the system-optimized value. Image *z*-stacks were deconvolved using the 3D deconvolution feature of LAS-X and its non-blind deblur algorithm with default system settings.

Measurements were collected on single plane images selected as being on the central line of each plane, and from several different planes in each stack. Surface areas of root epidermal cell planes were measured via multiple point polygons drawn to delineate the radial, transverse, and tangential planes using *z*-stack orthogonal projections (see Supplementary Fig. S1 for further explanation). Trichoblasts and atrichoblasts were identified by tracking the root hair through the *z*-stack to the epidermal cells of origin. Subsequently, trichoblasts and atrichoblasts were measured on tissues from the different recurrent selections for increased shoot vigour. Root hair density was assessed on genotypes from the two extreme cycles for vigour on these same images. A rectangle of 0.4 × 0.2 mm was placed over the centre of the root of each of the 10 samples per genotype. Trichoblasts in this rectangle were identified and counted and root hair density per mm^2^ was calculated. We define the ‘cell body’ of a trichoblast as the part of the root cell that is on the epidermal layer, excluding the protruding root hair (Supplementary Fig. S1A). Root hair diameter was measured at the base on 10 randomly chosen root hairs.

### Leaf cell imaging and measurements

Measurements of leaf epidermal cells used 2 cm sections of the fully expanded third leaf excised 2 cm from the base of the leaf. These were fixed in 70% ethanol for 24 h and transferred to 1% w/v bleach (White King© sodium hypochlorite 42 g l^–1^) for clearing over several days (Botwright *et al.*, 2005). Leaf pieces were mounted whole in water. Brightfield images were obtained using a Zeiss AxioImager Z1 microscope equipped with a Zeiss Axiocam 712 colour CCD camera (Carl Zeiss Micro-imaging GmbH, Jena, Germany) and plan-apochromat ×10 (numeric aperture=0.3) objective. Tiled images were captured to record epidermal cell position along with cell length and width measurements using Zeiss ZEN V2.6 (Carl Zeiss Micro-imaging GmbH).

### Statistical analyses

A mixed linear model was fitted containing random components that identified the structure of the experimental design for each experiment: (i) pot position (row, column); and (ii) soil type. The ANOVA and estimation of least squares means was conducted considering genotypes as fixed effects using the function lm in ‘emmeans’ (Lenth *et al.*, 2020) in the R (4.03) (R Core Team, 2020) software package. Pairwise comparisons between genotype means were obtained using the pairs function in the ‘emmeans’ package. The correlation between root hair length and rhizosheaths, and the relationship between the width of each of the three leaves and rhizosheath were tested using a Pearson’s correlation with the ‘cor.test’ in R.

## Results

### Shoot vigour, leaf width, and cell file number

Individual leaf traits were analysed to verify that the recurrent wheat lines varied significantly for vigour as previously described. Average widths of the first three fully emerged leaves increased significantly (*P*≤0.01) from the C0 lines, the least vigorous cycle, to the C5 lines, the most vigorous cycle (Supplementary Fig. S2). Average width of the first leaf in the C5 lines was 22% greater, 43% wider in the second leaf, and 39% greater in the third leaf compared with the C0 lines. There was a small but significant (*P*≤0.01) reduction in average leaf width for leaves 1, 2, and 3 between C5 and C6.

Closer examination indicated that the total number of veins across the leaves increased with increasing leaf width (Fig. 1A). The C5 lines had 65% more veins across the third leaf than the C0 and C1 lines. The number of cell files between adjacent veins (12.5 ± 0.2, SE) was not significantly different between the different selection cycles. Therefore, as the number of veins on the wider leaves increased, the total number of cell files across the leaves also increased (Fig. 1B). Leaves of C5 had an average of 410 ± 14 cells files across their width whereas C0 that had only 226 ± 14 cell files. Leaf width was strongly correlated with the number of cell files (*r*=0.77) (Fig. 1C).

### Relationship between increased shoot vigour and root biomass

To investigate whether selection for shoot vigour affected root growth, shoot and root biomass of plants at growth stage Z14 were measured. Shoot biomass and root biomass increased between C0 and C6 of the recurrent selections (Supplementary Fig. S3A, B), and no clear relationship was noted between shoot vigour and root to shoot ratio (Supplementary Fig. S3C). Recurrent selection cycles C0 and C3 had root to shoot ratios that were not significantly different from one another. Similarly, the ratios for C1, C4, C5. and C6 were also not significantly different (*P*>0.05).

### Relationship between increased shoot vigour, rhizosheath size, and root hair length

**Fig. 2.**
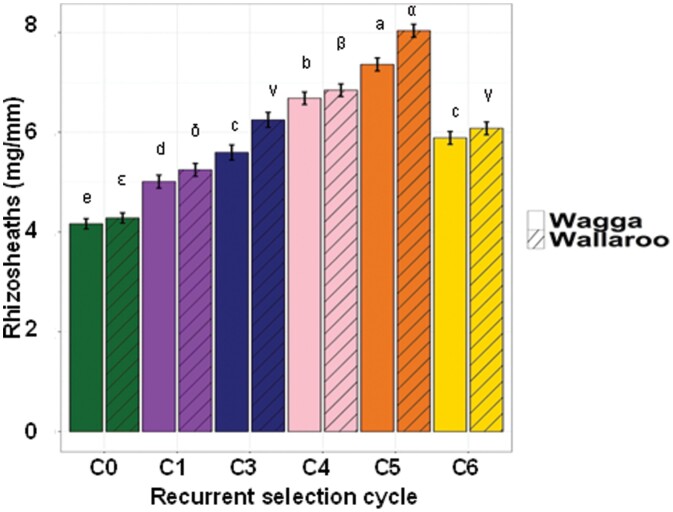
Changes in rhizosheath size with recurrent selection for shoot vigour. Data show the rhizosheath size on lines from each cycle of the recurrent selections for shoot vigour. Rhizosheaths were assayed in Podzol and Red Kandosol soils. Error bars indicate the SE (*n*=60 per soil type and cycle number). Note, the analyses for the two soils were performed separately with a one-way ANOVA as experiments with the two soils were undertaken separately and at different times. The Roman letters indicate significant differences (*P*≤0.05) for the Red Kandosol, and the Greek letters indicate differences for the Podzol soil.

**Fig. 3.**
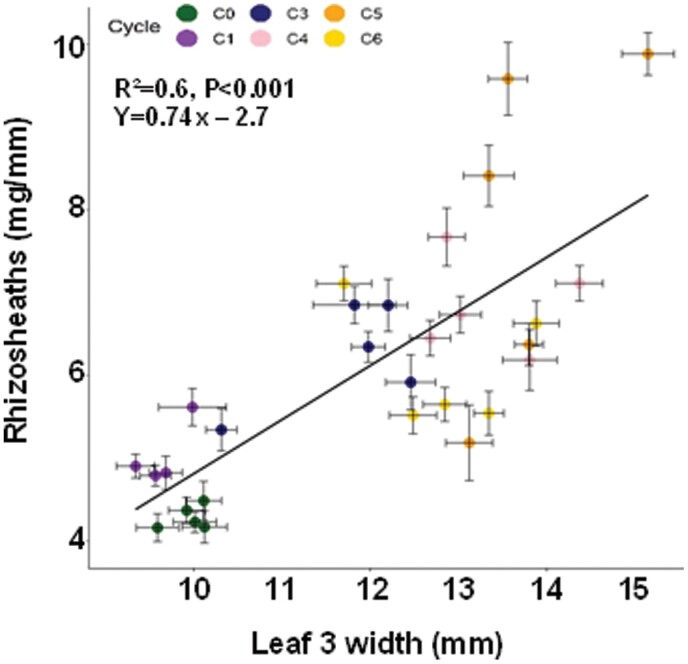
Relationship between shoot vigour and rhizosheath size. Rhizosheath size (mg mm^–1^ root length) was measured in the five lines from each of the recurrent selections and plotted against width of the third fully emerged leaf (shoot vigour). The error bars represent SEs for both leaf width and rhizosheaths (*n*=18 for rhizosheaths and *n*=24 for leaf width).

The seminal roots used to measure rhizosheath size had similar lengths across all cycles (Supplementary Fig. S). In contrast, rhizosheath size increased incrementally from the C0 to the C5 lines at ~17% per cycle, and then decreased (*P*<0.05) between C5 and C6 (Fig. 2). Average rhizosheath size in the C5 lines was 85% larger than in the C0 lines; however, C6 lines were 23% smaller than the C5 lines (*P*<0.05). Similar results were noted in experiments performed with both soils (Fig. 2), indicating that the differences in rhizosheath size were repeatable and robust. A significant correlation was detected between rhizosheath size and shoot vigour, with vigour assessed as width of the third leaf (Fig. 3).

**Fig. 4.**
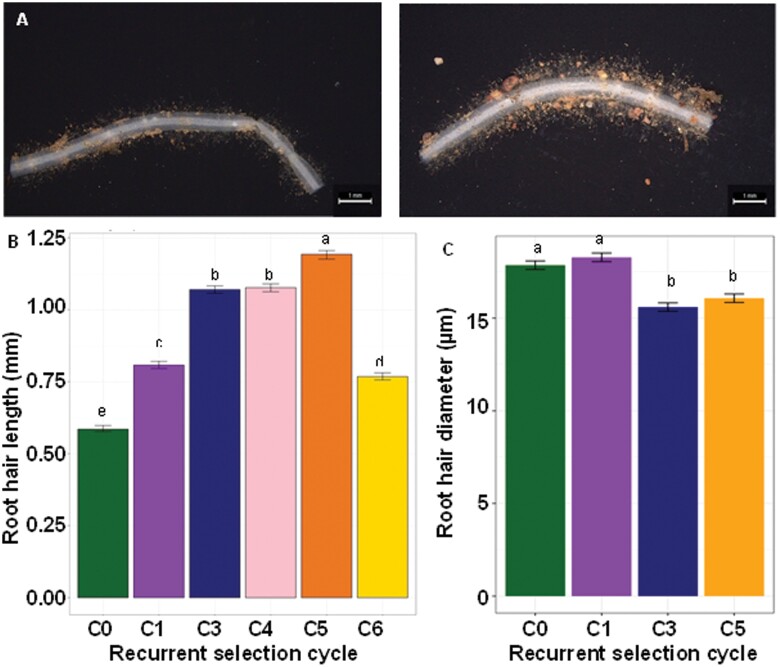
Influence of changes in shoot vigour on root hair length and diameter. The root tissues previously used for measuring rhizosheath size were rinsed of soil and preserved in 50% ethanol. The tissues were later photographed under the microscope. (A) Photographs of representative root segments taken from the seminal roots of C0 (left) and C5 (right) lines; scale bar=1 mm. (B) Root hair length of each of the recurrent selection cycles for increased shoot vigour (*n*=250 for each cycle). (C) Root hair diameter of a subsample of the recurrent selection cycles (*n*=250 for each cycle). The error bars represent SEs, and different letters identify significant differences in means at *P*=0.05.

To further confirm that rhizosheath size was a reliable surrogate for root hair length in the vigour germplasm, direct measurements of root hair length were made on a subset of the roots previously used to measure rhizosheath size (Fig. 4A). Significant differences (*P*<0.01) in root hair length were detected between lines from the recurrent selection cycles. Root hair length doubled from the least vigorous C0 lines to the most vigorous C5 lines (Fig. 4B), and a strong linear correlation occurred between root hair length and rhizosheath size (*r*=0.87, *P*<0.001). This demonstrates that rhizosheath size was a useful proxy for root hair length in this germplasm (Supplementary Fig. S5). Root hair diameters were also measured at the point where root hairs emerged from the trichoblast cells. The average root hair diameter in the C5 lines was 14% smaller than in the C0 lines (Fig. 4C).

### Increased shoot vigour is associated with smaller cell sizes and increased root hair density

**Fig. 5.**
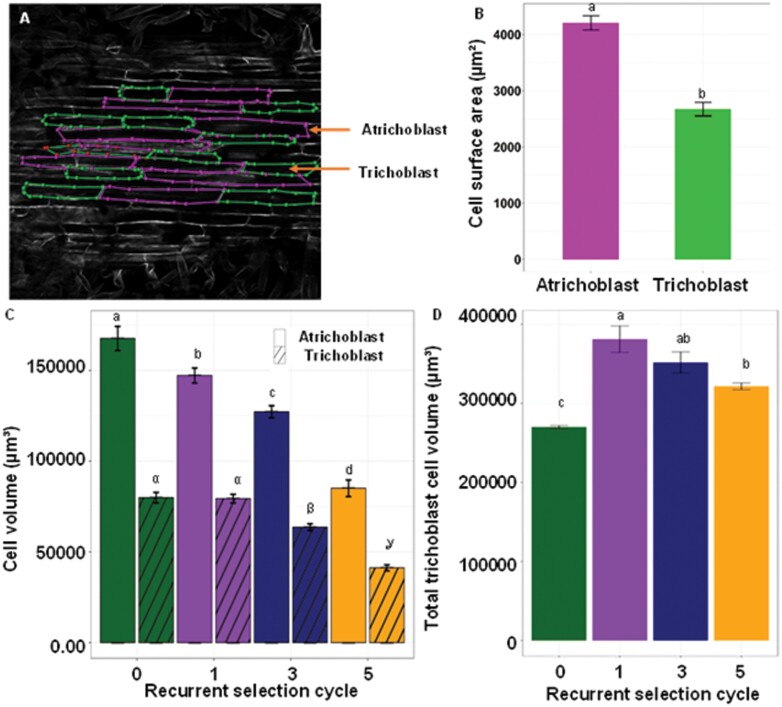
Influence of changes in shoot vigour on the size of root epidermal cells. (A) Single plane confocal microscopy image of root epidermal cells showing trichoblasts (green outline) and atrichoblasts (magenta outline). Trichoblasts were identified by changing the focus to identify those with root hairs. (B) Surface areas of the trichoblasts and atrichoblasts of C0 lines. The area measured was ‘surface 2’ of Supplementary Fig. S1 (*n*=64). (C) Cell body volumes of trichoblasts and atrichoblasts (excluding the root hair volume) for each cycle of the recurrent selection for shoot vigour (*n*=75 per cell type and cycle). (D) Total cell volumes of trichoblasts including root hair volume (*n*=75 per cell type and cycle). The error bars represent SEs, and different letters identify significant differences between means at *P*=0.05.

**Fig. 6.**
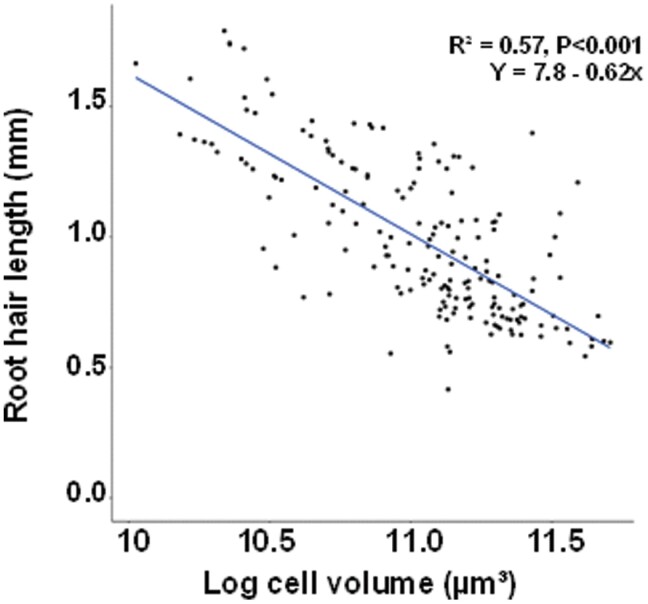
Relationship between trichoblast cell body volume and root hair length. Volumes of trichoblast bodies (transformed to the natural log) plotted against root hair length (*n*=180, samples issued from the rhizosheath experiment).

**Fig. 7.**
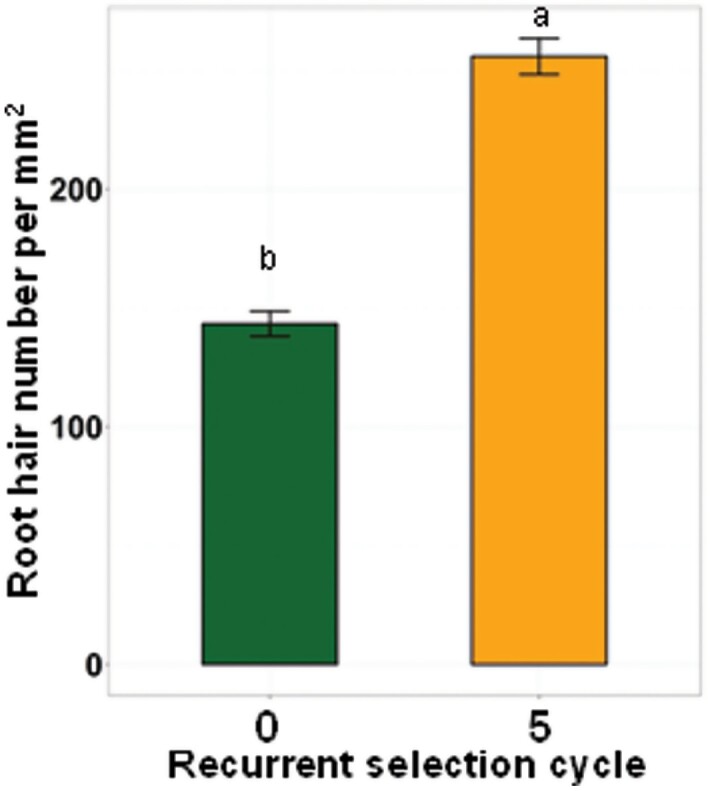
Influence of changes in shoot vigour on root hair density. Number of identified trichoblasts (cf Fig. 5A) per mm^2^ on roots of the two extreme cycles of the selection for increased shoot vigour. The error bars represent SEs, and different letters identify significant differences at *P*<0.05 (*n*=30).

To investigate how increased vigour affected cell size, confocal microscopy was used to measure epidermal cell size on the roots, and widefield microscopy was used to measure the size of leaf epidermal cells (Supplementary Fig. S1B). Figure 5A highlights the cell bodies of the trichoblasts and atrichoblasts on the root epidermis. The area of these cells was measured in each plane (Fig. 5A). These measurements initially focused on the body of the cells and not the protruding root hairs. Figure 5B shows that the atrichoblasts were larger than the trichoblasts, and this was consistent for all the vigour selections. The cell bodies of both trichoblasts and atrichoblasts decreased from C0 to C5 as shoot vigour increased. Volumes of both cell types in C0 (least vigorous) lines were almost double those of C5 (most vigorous) (Fig. 5C). Root hair length was negatively correlated with the natural log of trichoblast body volume (Fig. 6). The reduced body sizes of the trichoblasts and atrichoblasts in the high vigour lines was associated with a significant increase of root hair density as shown in Fig. 7. Indeed, C5 had 34% more root hairs per mm^2^ than the less vigorous C0.

Volumes of the protruding root hairs were then calculated from previous measurements of hair length and diameter (Fig. 4B, C). Total trichoblast volume was calculated by adding the hair volumes to the cell body volumes. Total trichoblast volumes were more similar across all recurrent selection cycles and did not show a consistent trend with shoot vigour (Fig. 5C). As shoot vigour increased, root hairs lengthened and largely compensated for the smaller volumes of the trichoblast body (Fig. 5D).

**Fig. 8.**
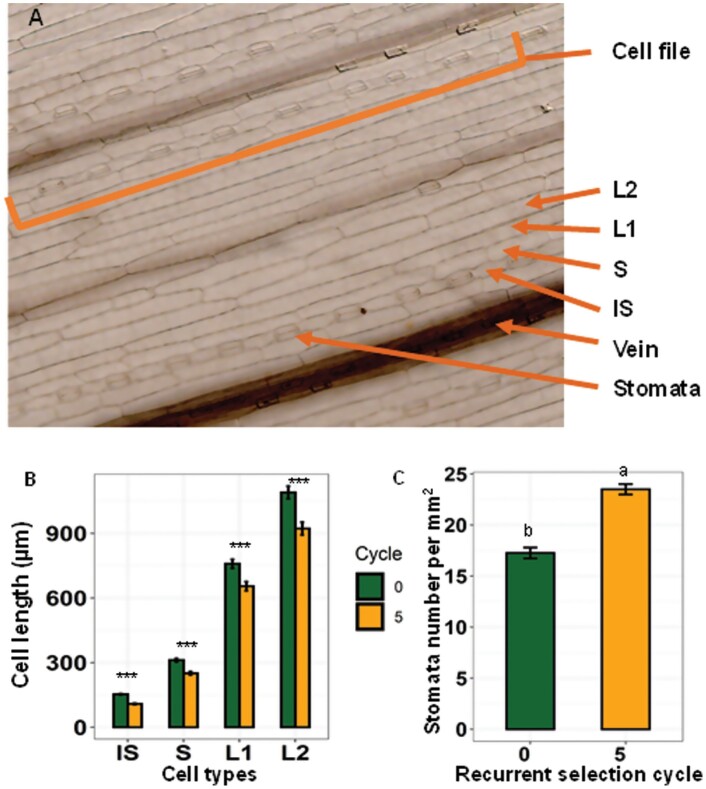
Influence of changes in shoot vigour on the size of leaf epidermal cells. (A) Abaxial leaf surfaces of a wheat leaf showing the four cell types measured including the interstomatal cells (IS), sister cells (S), elongated cell 1 (L1), and elongated cell 2 (L2), the veins and cell files of stomata and IS cells. (B) Lengths of the four cell types. Significance levels represent comparisons between C0 and C5 for each cell type. ∗*P*< 0.05, ∗∗*P*<0.01, and ∗∗∗*P*<0.001 (*n*=100 per cell type and cycle). (C) Average number of stomata observed per square millimetre. The error bars represent SEs, and different letters identify significant differences at *P*<0.05 (*n*=75).

To establish if increased shoot vigour is only associated with changes to the size of root cells, we also measured the dimensions of four cell types on the leaves of lines from C0 and C5, which represent the extremes for shoot vigour in the germplasm examined (Fig. 8A). The length of all four cell types was significantly shorter (*P*<0.001) for the high vigour C5 lines than the less vigorous C0 lines. Compared with C0, the interstomatal cells of C5 were 27% shorter, the sister cells were 20% shorter, and the elongated cell files 1 and 2 were 22% and 16% shorter, respectively (Fig. 8B). In contrast, the widths of these cell types were not significantly different among the selection cycles (*P*>0.05), except for the interstomatal cells, which were 6% wider (*P*<0.01). The differences in interstomatal cell size were associated with changes in stomatal density on the leaves such that the average stomatal density for the C5 lines was 36% greater than that of the C0 lines (Fig. 8C).

## Discussion

**Fig. 9.**
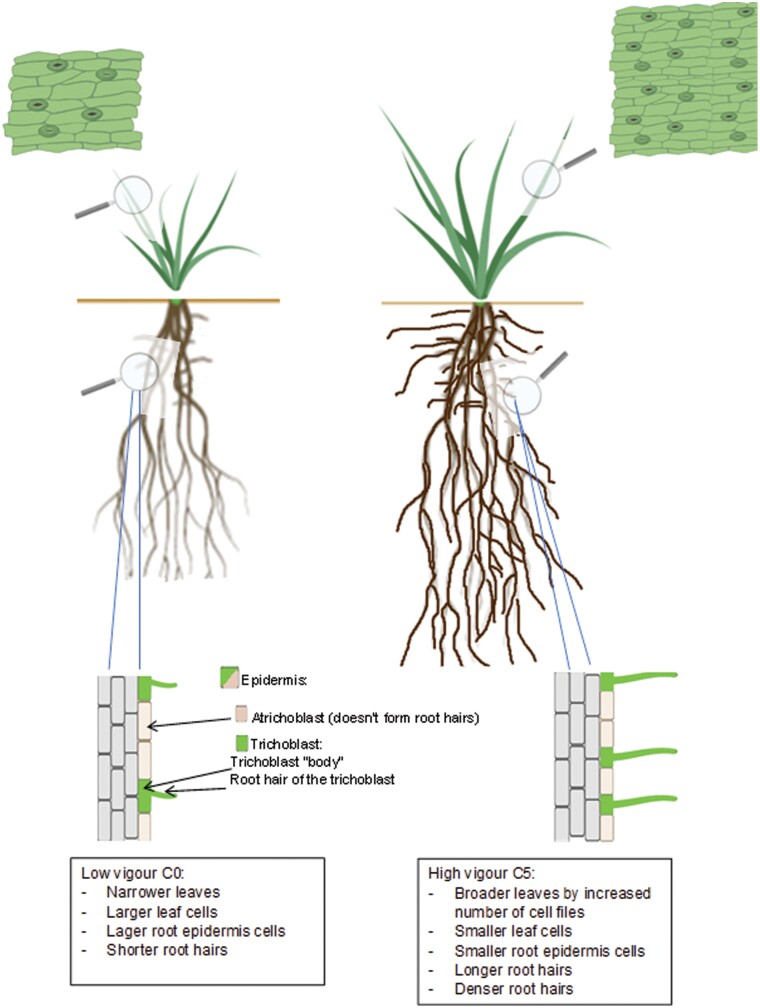
Diagram summarizing the impact of selection for shoot vigour on cells and organs.

This study used a novel wheat recurrent selection system expressing early vigour to examine whether changes to root and shoot cell properties were associated with early shoot vigour in progressive cycles of selection. Our results clearly demonstrate that recurrent selection for greater shoot biomass was strongly correlated with longer and denser root hairs and reduced cell sizes. Four different leaf cell types (interstomatal, sister, elongated 1, and elongated 2) (Fig. 8B) and two root epidermal cell types (trichoblasts and atrichoblasts) became progressively smaller as shoot vigour increased (Fig. 5C), indicating that the selection for early shoot vigour induced developmental changes throughout the plant. A summary of the developmental changes to shoots and roots associated with increased shoot vigour is provided in Fig. 9.

Interestingly, while the cell bodies of trichoblasts on the roots of the high vigour lines were smaller than those on the less vigorous lines (Fig. 5C), the root hairs were significantly longer so the total trichoblast volume (combination of cell body and protruding root hair) showed no consistent relationship with shoot vigour (Fig. 5D). A similar inverse relationship between root hair length and trichoblast body size was recently reported in Arabidopsis (Sánchez-Fernández *et al.*,1997; Chen *et al.*, 2015; Canales *et al.*, 2017). Turgor pressure is an important driving force for hair elongation in trichoblasts (Mendrinna and Persson, 2015; Haas *et al.*, 2020) along with cell wall composition and specific enzymes that contribute to wall loosening (Cosgrove, 2005; Winship *et al.*, 2010; Kozlova *et al.*, 2014; Zhang and Zhang, 2020). Turgor tends to be greater in smaller plant cells (Long *et al.*, 2020), and higher turgor in the smaller trichoblasts could be the driving force for the development of longer root hairs in high vigour lines.

A corollary to the smaller epidermal cell size in high vigour lines is that the density of root hairs becomes significantly greater with high vigour, and we demonstrated this with direct measurements (Fig. 7). Therefore, not only do more vigorous lines have longer root hairs, they also exhibit greater density of root hairs than less vigorous lines. This is consistent with previous findings by Gahoonia *et al.* (1997) who demonstrated that wheat genotypes with longer root hairs also possessed more root hairs per unit area. In our study, the cell body sizes of the trichoblasts were consistently smaller than those of the atrichoblasts across all selection cycles (Fig. 5B). Asymmetry between trichoblasts and atrichoblasts was originally observed in *Brassicae* plants (Cormack, 1935). Our results are consistent with early observations in the *Poacea* by Row and Reeder (1957) and more recent reports in barley, *Brachypodium distachyon*, and rice (*Oryza sativa* L.) (Kim and Dolan, 2011; Marzec *et al.*, 2013).

The size of the leaf epidermal cells also decreased as shoot vigour increased (Fig. 8A), and stomatal density was 36% greater in the high vigour lines compared with the less vigorous lines (Fig. 8B). Changes in stomatal density probably impact photosynthesis and transpiration, and may partly explain why early shoot vigour appears to stabilize yields in regions with variable rainfall (Wilson *et al.*, 2015). Previous studies investigating the link between shoot vigour and leaf anatomy have reported contradictory findings. In agreement with the results presented here, Ter Steege *et al.* (2005) reported that early vigour in *Aegilops tauschii* was positively correlated with cell number and negatively correlated with maximum cell length. In contrast, increased vigour in ryegrass reportedly increased cell size and volume (Smith *et al.*, 2003). Hybrid vigour in Arabidopsis was similarly associated with larger leaves and an increase in cell size (Greaves *et al.*, 2015; Fujimoto *et al.*, 2018). In wheat, Botwright *et al.* (2005) determined that one out of the four leaf cell types examined (‘long’ cells) was longer in a vigorous genotype while all other cell types did not differ in size. However, it should be emphasized that the vigorous germplasm examined by Botwright *et al.* (2005) was Vigour 18 which was one of the least vigorous lines included in C0 of the present study.

The wider leaves in the high vigour lines possessed significantly more veins and cell files across their width. Wider leaves have been linked to an increase in vein number in barley (Thirulogachandar *et al.*, 2017) and with more cell files in wheat (Botwright *et al.*, 2005; Spielmeyer *et al.*, 2007). Vein number is an anatomical feature associated with plant performance because vein density can also impact photosynthetic efficiency. An increase in cell file number without a proportional increase in leaf vein number can negatively impact transport processes and photosynthesis (Feldman *et al.*, 2017). We observed that leaf widths increased from C0 to C5 cycles, but significantly decreased from C5 to C6. Interestingly, using similar germplasm, Zhang *et al.* (2015) showed that leaf width remained stable in C5 and C6 lines. Our experiments used a subsample of the original genotypes assessed by Zhang *et al.* (2015) (five of 30 lines), which may account for these contrasting results. Nevertheless, an important finding to emerge from the germplasm examined in the present study was the strong positive associations between leaf width, leaf vein number, and leaf cell file number. In our study, root to shoot ratios remained relatively stable as shoot vigour increased, suggesting that carbon allocation between roots and shoots was generally conserved regardless of changes to vigour. This is consistent with previous conclusions using some of the progenitors of the germplasm examined in the present study (Palta *et al.*, 2011; Palta and Yang, 2014).

This study confirmed that rhizosheath size is a reliable surrogate for root hair length in wheat (Delhaize *et al.*, 2012, 2015). Rhizosheath size showed an average increase of 17% per selection cycle from C0 to C5 which is more than double the increase in leaf width of 7.3% per cycle described by Zhang *et al.* (2015). Indeed, as root hair length nearly doubled from C0 to C5, leaf width increased only by 35%. Nevertheless, a strong positive correlation was detected between shoot vigour (leaf width) and root hair length (rhizosheath size) across all lines.

Interestingly, both root hair length and shoot vigour in wheat are described as complex quantitative traits (Coleman *et al.*, 2001; Horn *et al.*, 2016). The surprising finding that these traits co-segregated with one another during the six cycles of recurrent selection for shoot vigour has two possible explanations. The first explanation is that genes controlling these two independent traits are closely linked and their co-selection was a consequence of their genetic linkage. A second explanation is that the genes controlling shoot vigour have pleotropic effects on root hair length. It is intriguing to consider the mechanisms that could link shoot vigour with root hair length, but the significant correlations observed between shoot vigour and cell size in the roots and the leaves clearly point to the fact that changes to whole-plant development have occurred.

Recent studies have shown that vigorous wheats are generally more competitive against weeds, and this is largely attributed to their ability to generate early biomass, intercept more light, and shade-out competitors (Mwendwa *et al.*, 2020). We established here that high vigour lines also have larger root systems, longer and denser root hairs, and denser stomata, all of which could enhance soil exploration and increase the uptake of water and nutrients (Gilroy and Jones, 2000; Rongsawat *et al.*, 2021). There appear to be surprising links between root hair growth and stomatal density, some of which have been reported previously. For instance, Hepworth *et al.* (2016) examined a range of transgenic Arabidopsis lines with altered stomatal patterning and reported a positive correlation between stomatal density and total root area, root hair density, and root hair length. While the mechanism for these relationships remains unclear, the authors suggested that increasing stomatal density increased transpiration and the demand for water which then stimulated root hair growth. However, in another Arabidopsis mutant carrying targeted knockouts in genes known to affect root hair growth, the authors found a negative relationship between root hair density and stomatal density. These results are intriguing, and the links between guard cell patterning and root hair growth deserve further attention.

The greater root hair length and density of the vigorous wheat lines is likely to improve competitiveness by facilitating the acquisition of essential resources (Craine and Dybzinski, 2013), particularly for plants grown under constrained environments as found for barley (Marin *et al.*, 2021). For example, Haling *et al.* (2013) found that root hairs improved the ability of roots to penetrate soil, increased the root to soil contact, and enhanced P acquisition. Improved root penetration was evident in high strength soils (Haling *et al.*, 2013) and in soils with variable densities (Bengough *et al.*, 2016) where the hairs were thought to provide sufficient tensile strength to anchor root apices. These root traits might provide additional advantages to plants because they are also important for the release of both primary and secondary metabolites including allelochemicals (Yan *et al.*, 2004; Weston *et al.*, 2012). For example, in sorghum (*Sorghum* ssp.), root hairs have been identified as the sole site for the exudation of the allelochemical sorgoleone (Czarnota *et al.*, 2003). In barley, hairless mutants were shown to exude three times less carbon into the soil compared with the wild type (Holz *et al.*, 2018). In wheat, roots and rhizosphere processes are important for the release and transformation of benzoxazinones and phenoxazinones (Mwendwa *et al.*, 2021). Since the more vigorous wheat lines had larger root systems with longer and denser root hairs, it is reasonable to speculate that they may also exude a greater amount of carbon into the rhizosphere, which may include allelochemicals useful for the suppression of competing species (Venturelli *et al.*, 2015; Niculaes *et al.*, 2018; Mwendwa *et al.* 2021).

### Conclusion

This study demonstrated that recurrent selection for increased shoot vigour had pleotropic effects on other aspects of plant anatomy and growth. Greater shoot vigour was significantly correlated with larger root systems and with longer root hairs. Moreover, shoot vigour was linked with smaller epidermal cells on the roots and leaves which increased the density of root hairs and stomata. We conclude that the superior competitiveness of vigorous wheat lines could, in part, be explained by root traits that enhance their ability to acquire essential resources. Future work will investigate the involvement of hormones in early vigour and whether the exudation of allelopathic compounds could also contribute to the competitiveness of vigorous germplasm.

## Supplementary data

The following supplementary data are available at *JXB* online.

Fig. S1. Influence of changes in shoot vigour on the size of root epidermal cell body.

Fig. S2. Leaf width of the cycles of the recurrent selection for shoot vigour.

Fig. S3. Influence of changes in shoot vigour on plant biomass and root to shoot ratio.

Fig. S4. Changes in root length with recurrent selection.

Fig. S5. Relationship between root hair length and rhizosheath size.

erac048_suppl_Supplementary_Figures_S1-S5

## Data Availability

The data supporting the findings of this study are available from the corresponding author, P.-W. Hendriks.
