# Supplementary material for: Selection for early shoot vigour in wheat increases root hair length but reduces epidermal cell size of roots and leaves
Source: J Exp Bot. 2022 Feb 23;73(8):2499–510. doi: 10.1093/jxb/erac048 (PMC9015806; doi:10.1093/jxb/erac048)
Supplement: erac048_suppl_Supplementary_Figures_S1-S5 [file erac048_suppl_supplementary_figures_s1-s5.pdf]

Supplementary data

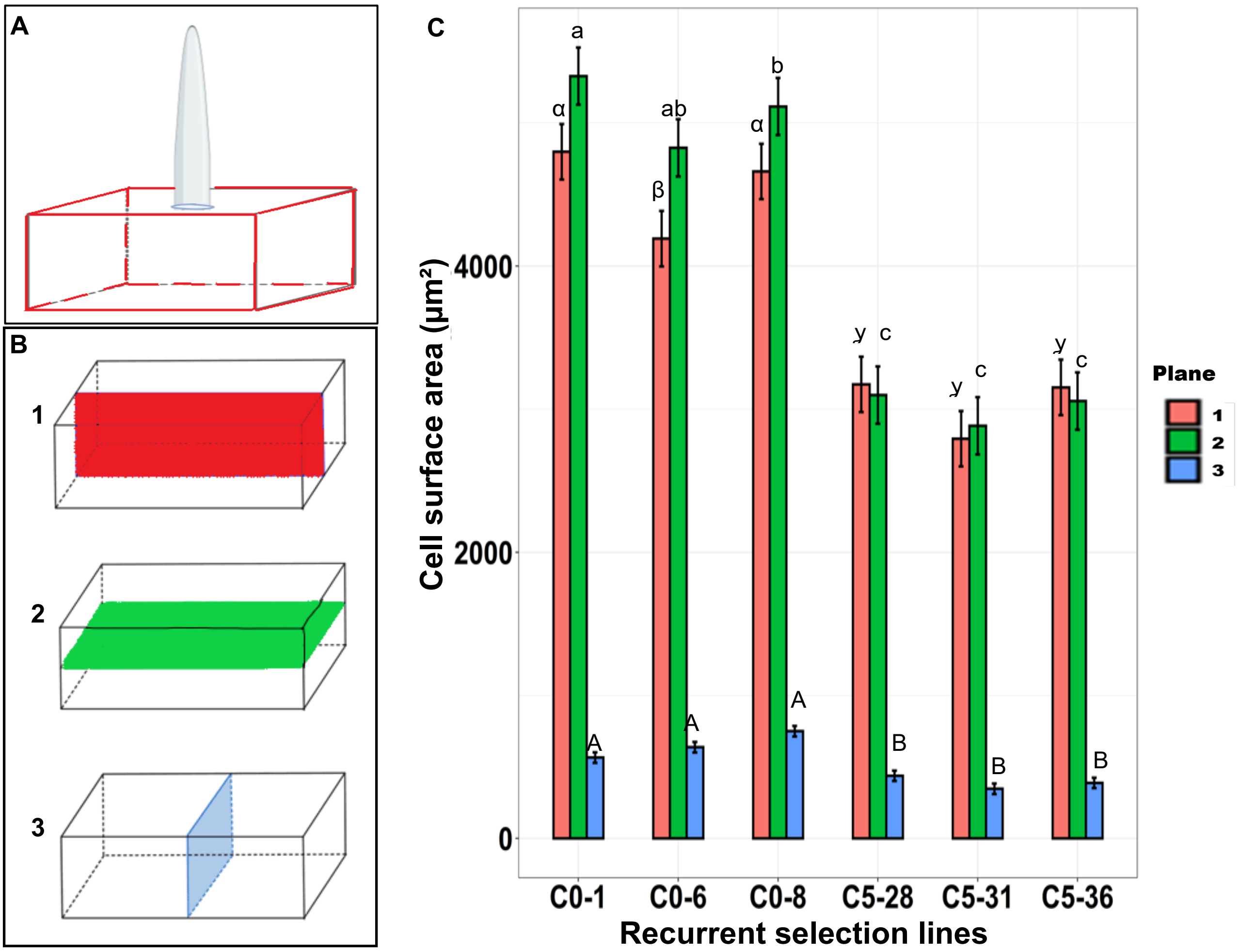

Fig. S1: Influence of changes in shoot vigour on the size of root epidermal cell body. A) Diagram of a single trichoblast with the "cell body" outlined in red and the root hair in grey. B) Representation of the surface area measurements collected on three planes of the root epidermal cells. C) Calculated surface areas of these planes on three different lines from the C0 selection cycle (least vigorous) and from three lines from the C5 selection cycle (more vigorous). The error bars represent standard errors (n = 100). Letters identify significant differences at $P < 0.05$ for each category of leaves (a,b,.. for cross-section 1; α,β,.. for cross-section 2 and A,B,.. for cross-section 3).

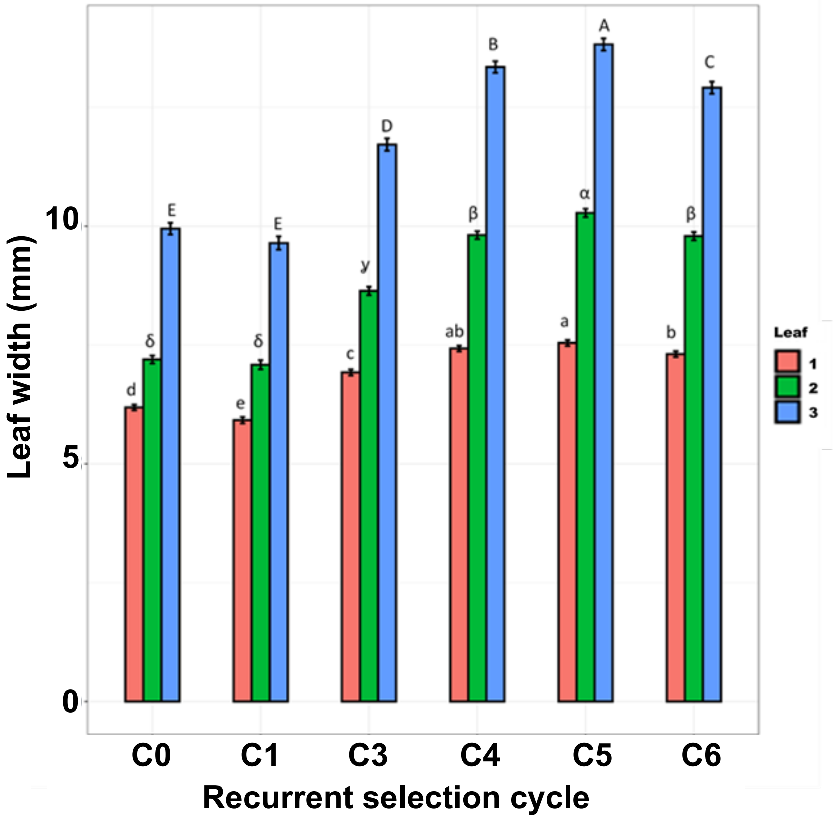

Fig. S2: **Leaf width of the cycles of the recurrent selection for shoot vigour.**

The figure shows the widths of the first three fully-expanded leaves of lines from each cycle of the recurrent selections for shoot vigour. Error bars represent standard errors (n=60 per leaf and cycle). Letters identify significant differences at $P < 0.05$ for each category of leaves (a,b,.. for leaf 1; α,β,.. for leaf 2 and A,B,.. for leaf3). Different colours represent the first (red), second (green) and third (blue) leaves.

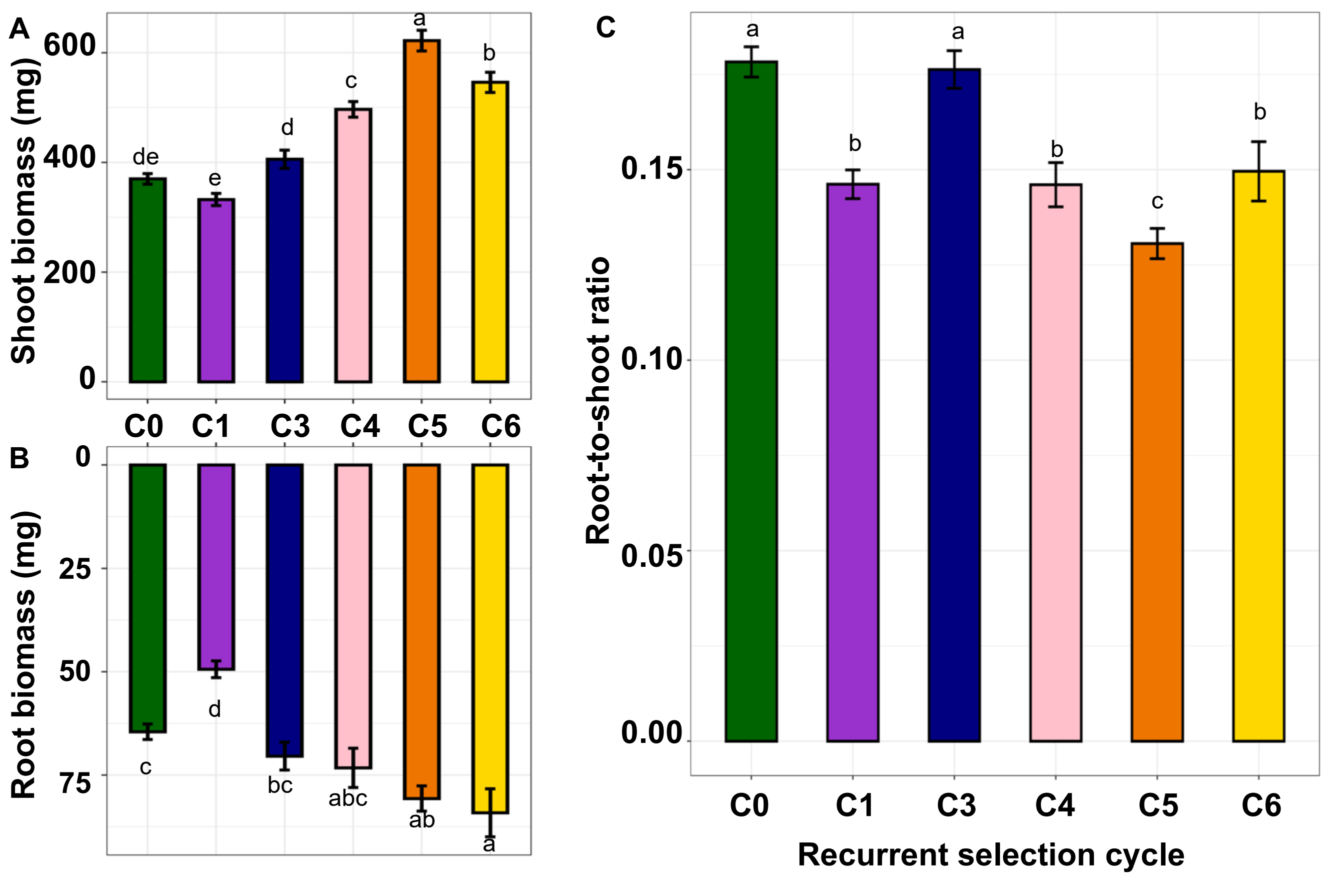

Fig. S3: **Influence of changes in shoot vigour on plant biomass and root-to-shoot ratio.**

Results shown are A) shoot biomass, B) root biomass and C) root-to-shoot ratio of the different cycles of recurrent selection. Root and shoot biomass were measured at Z14 when the fourth leaf had fully emerged. The error bars represent standard errors (n=24 per cycle), and the different letters identify significant differences at P = 0.05.

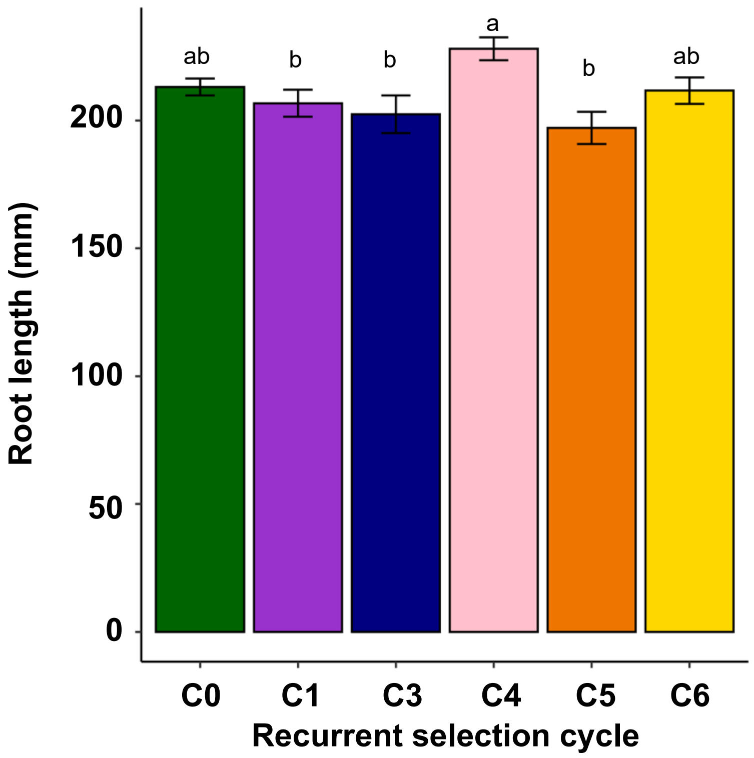

Fig. S4: Change in root length with recurrent selection.

Results show the combined lengths of the three seminal roots from the eight-day old seedings used to measure rhizosheath sizes. The error bars represent standard errors (n=60), and the different letters identify significant differences at P = 0.05.

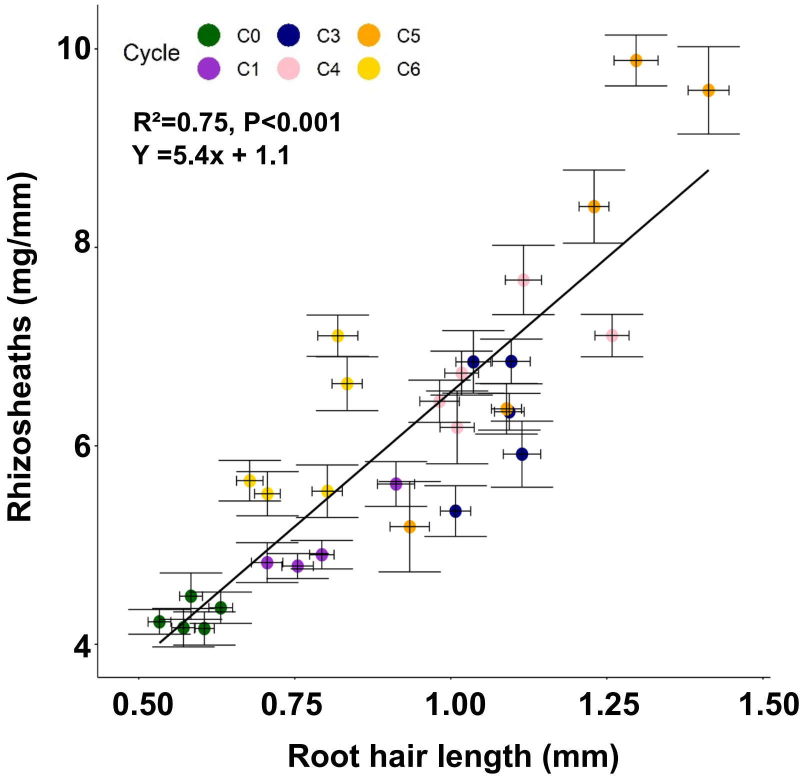

Fig. S5: Relationship between root hair length and rhizosheath size. Roots of seedlings comprising of five genotypes per cycle of the recurrent selection were assayed as described in Materials and Methods. The error bars represent standard errors (n=18).
